# DFT-guided Lennard–Jones parametrization for accurate $\text {CO}_{2}$, $\text {N}_{2}$, and $\text {CH}_{4}$ adsorption in MoOFOUR-1-Ni

**DOI:** 10.1007/s00894-026-06866-6

**Published:** 2026-08-01

**Authors:** Herick Ribeiro Torres, Roberta Pereira Dias, Heitor Avelino de Abreu, Júlio Cosme Santos da Silva, Guilherme Ferreira de Lima

**Affiliations:** 1 Departamento de Química, Universidade Federal de Minas Gerais, Av. Antônio Carlos, 6627, Pampulha, Belo Horizonte, 31270-901 MG Brazil; 2 Instituto de Química e Biotecnologia, IQB, Universidade Federal de Alagoas, Campus A. C. Simões, Maceió, 57072-900 Al Brazil

**Keywords:** APMOFs, Force field parametrization, Grand canonical monte carlo, Natural gas separation, Lennard–Jones potential

## Abstract

**Context:**

Anion-pillared metal–organic frameworks (APMOFs) have emerged as promising materials for natural gas purification, owing to the strong electrostatic fields generated by high-charge-density ionic pillars. However, accurately describing these highly localized interactions within classical force field frameworks remains challenging, as generic parameterizations such as the Universal Force Field (UFF) and DREIDING fail to capture the specific polarization effects of ionic active sites. In this work, we address this limitation for MoOFOUR-1-Ni, an APMOF exhibiting high CO$_{{\textbf {2}}}$ selectivity over N$_{{\textbf {2}}}$ and CH$_{{\textbf {4}}}$, by developing a system-specific force field for the $[\text {MoO}_{{\textbf {4}}}]^{{\textbf {2}}-}$ pillar through a DFT-guided parametrization workflow. The optimized Lennard–Jones parameters were validated against experimental adsorption isotherms for all three gases, yielding a five-fold reduction in mean absolute error for CO$_{{\textbf {2}}}$ relative to UFF+DREIDING, and very good agreement for N$_{{\textbf {2}}}$ and CH$_{{\textbf {4}}}$. The results highlight the inherent substrate-specificity of the derived parameters and the fundamental limitations of transferable force fields in chemically complex ionic environments.

**Methods:**

Potential energy curves for the interactions between the $[\text {MoO}_{{\textbf {4}}}]^{{\textbf {2}}-}$ anion and CO$_{{\textbf {2}}}$, N$_{{\textbf {2}}}$, and CH$_{{\textbf {4}}}$ were computed at the PBE-D3(BJ)/def2-TZVPD level of theory using ORCA 6.0.1. Partial charges were obtained via the CHELPG method as implemented in Multiwfn. Lennard–Jones parameters were optimized using the L-BFGS-B algorithm, minimizing a regularized objective function combining mean absolute deviation from DFT reference energies and a quadratic penalty term. Grand Canonical Monte Carlo (GCMC) adsorption isotherms were simulated using RASPA on a $\boldsymbol{2\times 2\times 2}$ supercell derived from a PBE-D3/Quantum ESPRESSO periodic DFT optimization, with TraPPE parameters employed for the adsorbate molecules. Pure-component adsorption isotherms were further fitted to Langmuir-based models and employed in Ideal Adsorbed Solution Theory (IAST) calculations to estimate binary CO$_{{\textbf {2}}}$/CH$_{{\textbf {4}}}$ and CO$_{{\textbf {2}}}$/N$_{{\textbf {2}}}$ adsorption selectivities.

**Graphical abstract:**

**Figure:**
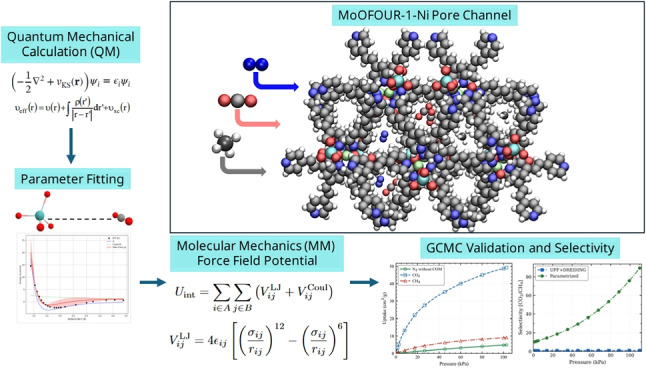

**Supplementary Information:**

The online version contains supplementary material available at 10.1007/s00894-026-06866-6.

## Introduction

Natural gas is a critical pillar of the global energy sector and an important feedstock for the chemical industry, being methane ($\textrm{CH}_{4}$) its main component comprises around 70–90% [1, 2]. Furthermore, it represents a more sustainable alternative to heavy petroleum derivatives and mineral coal, primarily due to its lower carbon dioxide ($\textrm{CO}_{2}$) emissions [3, 4]. In addition to heavier hydrocarbons such as ethane ($\textrm{C}_{2}{\textrm{H}}_{6}$) and propane ($\textrm{C}_{3}{\textrm{H}}_{8}$), raw natural gas contains considerable amounts of non-combustible gases such as nitrogen ($\textrm{N}_{2}$) and $\textrm{CO}_{2}$ [5]. The concentration of these components directly affects the final heating value and overall energy conversion efficiency [6–8]. To meet international regulations, $\mathrm {N_2}$ must be maintained below 1% for the transport of liquefied natural gas (LNG), and between 3% and 4% for pipeline distribution (as stipulated by ISO 13686) [9, 10]. Beyond decreasing the calorific value, the presence of $\textrm{CO}_{2}$ in natural gas compromises infrastructure by promoting equipment corrosion and causing blockages in the presence of moisture. For this reason, its removal is a mandatory pre-treatment step for natural gas liquefaction [11]. Furthermore, while $\textrm{C}_{2}{\textrm{H}}_{6}$ and $\textrm{C}_{3}{\textrm{H}}_{8}$ are treated as impurities within natural gas streams, they are highly valuable feedstocks for olefin production, and their recovery from natural gas is desired [2].

Natural gas purification is challenging because of the similar physicochemical properties of its components. For example, the kinetic diameters of $\mathrm {CO_2}$, $\textrm{N}_{2}$, and $\textrm{CH}_{4}$ are 3.30, 3.64, and 3.76 $\mathring{\textrm{A}}$, respectively [12]. Consequently, achieving effective separation solely via molecular sieving is highly challenging [13]. Therefore, conventional technologies involve cryogenic distillation, amine absorption, and pressure swing adsorption (PSA) techniques [14]. However, these traditional technologies suffer from severe energy penalties and high capital costs, thereby imposing significant limitations on their widespread deployment [15, 16].

To overcome the limitations of established methods, extensive research has been directed toward new advanced porous materials [17]. Among these, Metal-Organic Frameworks (MOFs) have emerged as promising candidates. MOFs are porous materials whose properties can be adjusted by an adequate selection of building blocks. Guided by the pioneering concept of reticular chemistry proposed by Yaghi [18], both the pore dimension and chemical environment of the cavities can be systematically engineered [19]. Within a broad spectrum of potential applications, the utilization of these frameworks for separation process stands out promising, especially for challenging gas mixtures comprised of molecules with nearly identical properties [20–22].

Considering the MOF materials, a notable subclass is the anion-pillared MOFs (APMOFs), which are 2D MOFs pillared by anions such as $\textrm{SiF}_6^{2-}$, $\textrm{NbOF}_5^{2-}$, $\textrm{BF}_4^{-}$, and $\textrm{MoO}_4^{2-}$. Beyond the possibility of tuning the pore size by choosing appropriate building blocks, the anions induce a very strong electrostatic field inside the pores, enhancing the contribution of both electrostatic and hydrogen bond interactions with guest molecules [23]. Specifically, exploiting the electric field in the cavity is a possible pathway for the separation of natural gas components, since the nonpolar $\textrm{CO}_{2}$, $\textrm{N}_{2}$, and $\textrm{CH}_{4}$ have quadrupole moment of -4.30, -1.52, and $0 \times 10^{-26} \text { esu} \, \text {cm}^{2}$, respectively [24].

To fully harness the principles of reticular chemistry for the design of advanced materials for challenging gas separations, a fundamental understanding of these host-guest interactions at the molecular level is critical. While DFT calculations, often coupled with topological analyses of the electron density, provide profound insights into the fundamental nature and strength of gas molecule-material interactions [1, 25–30], they are inherently limited by their high computational cost. Consequently, standard static DFT approaches struggle to model the competitive adsorption of multiple guest molecules or to encompass the dynamic and entropic effects that govern macroscopic separation.

To overcome these limitations, classical Molecular Dynamics (MD) and Monte Carlo (MC) simulations are methods that offer valuable insights into the spatial distribution and diffusion behavior of guest molecules within the cavities. In particular, Grand Canonical Monte Carlo (GCMC) simulations enable the direct computation of adsorption isotherms, providing macroscopic theoretical predictions that can be directly validated against experimental data [31].

Nevertheless, the quality of classical simulations is highly dependent on the force field, and generic force fields such as the Universal Force Field (UFF) [32] and Dreiding [33] often struggle to capture the highly specific polarization effects and dense electrostatic fields confined within MOF cavities [34]. Part of this limitation arises from the fact that widely used force field approaches, such as those based on the UFF or DREIDING parameters, prioritize transferability over the level of accuracy required to describe complex systems with specific interaction sites or structural defects [35, 36]. In fact, MOF materials frequently feature metal ions with vastly different chemical environments [29, 37], including the presence of open metal sites [38]. Describing this heterogeneous landscape with a single, universal set of parameters becomes fundamentally inadequate. For example, Li et al. [39] and Cho et al. [40] have shown that UFF overestimates the short-range repulsive interaction, thereby distorting the predicted adsorption isotherms. Furthermore, Brabson et al. [27] demonstrate that classical force fields fail to describe the guest-induced deformations in the MOF. Other studies have also reported that these force fields frequently and substantially underestimate the interaction strength between adsorbates and open metal sites commonly present in many metal–organic frameworks [35, 41, 42].

To overcome the limitations of generic force fields, customized parameterization, typically derived from high-level quantum chemical calculations of MOF–adsorbate interaction energies, is essential. In this context, MoOFOUR-1-Ni stands out as a particularly demanding and relevant target system. This anion-pillared MOF has been reported to exhibit high selectivity for CO$_2$ over N$_2$ and CH$_4$ even at low pressures [43], a behavior attributed to the strong electrostatic field generated by the [MoO$_4$]$^{2-}$ pillars. However, accurately capturing this highly localized and anisotropic interaction environment within a classical force field framework poses a significant challenge, as generic parameters are not designed to resolve the specific polarization effects of high-charge-density ionic sites. Herein, we detail a step-by-step computational workflow to derive a robust, system-specific force field capable of capturing the unique electrostatic landscape of the MoOFOUR-1-Ni cavities. By accurately parameterizing the electrostatic and van der Waals interactions from *ab initio* calculations, we provide a highly accurate microscopic description of the adsorption and separation behavior of natural gas components (CO$_2$, N$_2$, and CH$_4$) within this complex porous environment.

## Computational details

### DFT interaction energy calculations

The Lennard–Jones parameters (ϵ and σ) of the molybdate anion in the MoOFOUR-1-Ni structure were determined from potential energy curves describing the interactions between the $\textrm{MoO}_4^{2-}$ anion and gas molecules of interest ($\textrm{CO}_{2}$, $\textrm{N}_{2}$, and $\textrm{CH}_{4}$). To this end, three distinct systems were constructed, each consisting of the $\mathrm {MoO_4^{2-}}$ anion and one gas molecule ($\textrm{CO}_{2}$, $\textrm{N}_{2}$, or $\textrm{CH}_{4}$). The molybdenum atom was fixed at the origin of the coordinate system, while the gas molecule was placed at varying distances from the molybdenum center.

Energy data were obtained from DFT calculations performed using the ORCA program (version 6.0.1) [44], employing the PBE functional [45] in combination with the def2-TZVPD basis set (Triple-Zeta Valence Polarized with diffuse functions), developed by Ahlrichs [46], along with Grimme’s D3 dispersion correction with Becke–Johnson damping (D3(BJ)) [47]. Partial charges were computed using the CHELPG method [48], as implemented in the Multiwfn software [49]. The resulting atomic charges for the [MoO$_4]^{2-}$ anion are reported in Table S1; a representative ORCA input file illustrating the scan protocol is provided in Section S3 of the Supplementary Information.

To obtain the potential energy as a function of the interfragment distance, single-point calculations were performed with all geometries kept fixed. The interaction energy was computed according to Eq. 1, where $U_{\text {system}}$, $U_{\textrm{MoO}_4^{2-}}$, and $U_{\text {molecule}}$ denote the total energy of the interacting system, the isolated anion, and the gas molecules, respectively.

1 $$\begin{aligned} \Delta U = U_{\text {system}} - \left( U_{\mathrm {MoO_4^{2-}}} + U_{\text {molecule}} \right) \end{aligned}$$

### Force field parametrization

Based on the computed interaction energies, the data were fitted using a combination of Lennard–Jones and Coulomb potentials.

The input dataset consisted of sequential frames containing the number of atoms in each fragment, two reference atom indices used to define the intermolecular distance, the DFT interaction energy, and atom-resolved information including Cartesian coordinates, partial charges, and initial LJ parameters ϵ and σ. Initial atom-type parameters for Mo, O, C, N, and H were taken from the corresponding ϵ and σ values of the UFF force field supplied in the input structures.

For each geometry, the interaction energy was evaluated as a double sum over all interfragment atom pairs according to Eq. 2:

2 $$\begin{aligned} U_{\textrm{int}}= \sum _{i \in A}\sum _{j \in B} \left( V_{ij}^{\textrm{LJ}} + V_{ij}^{\textrm{Coul}} \right) \end{aligned}$$

where the Lennard–Jones term was defined as

3 $$\begin{aligned} V_{ij}^{\textrm{LJ}}= 4\epsilon _{ij} \left[ \left( \frac{\sigma _{ij}}{r_{ij}}\right) ^{12} - \left( \frac{\sigma _{ij}}{r_{ij}}\right) ^{6} \right] \end{aligned}$$

and the Coulombic contribution as

4 $$\begin{aligned} V_{ij}^{\textrm{Coul}} = 332.06371\, \frac{q_i q_j}{\epsilon _r \, r_{ij}} \end{aligned}$$

with ${r}_{ij}$ in Å, charges in units of the elementary charge, and energies in kcal mol$^{-1}$. A relative dielectric constant of $\epsilon _r = 10.0$ was used throughout. The choice of $\epsilon _r = 10.0$ ensures numerical stability during the parameter optimization process. In a vacuum ($\epsilon _r = 1.0$), the magnitude of the Coulombic forces can become disproportionately large, effectively overwhelming the Lennard–Jones potential terms. This imbalance often leads the optimization algorithm toward unphysical local minima characterized by unrealistic interatomic distances. By scaling the electrostatic contributions, the potential energy surface becomes more balanced, allowing for a more robust refinement of the van der Waals parameters against the DFT reference data. This effective dielectric screening is a common approximation in non-polarizable force fields to implicitly incorporate the electronic response of the medium that is not explicitly described by fixed atomic charges [50–52].

Mixed LJ parameters were generated using Lorentz–Berthelot combining rules,

5 $$\begin{aligned} \sigma _{ij}= &   \frac{\sigma _i+\sigma _j}{2} \end{aligned}$$

6 $$\begin{aligned} \epsilon _{ij}= &   \sqrt{\epsilon _i\epsilon _j} \end{aligned}$$

so that the total interaction energy at each scan point reflects the balance between short-range repulsion/dispersion and long-range electrostatic contribution.

The optimization strategy was designed to refine only the LJ parameters associated with the molybdate fragment. Accordingly, $\epsilon _{\textrm{Mo}}$, $\sigma _{\textrm{Mo}}$, $\epsilon _{\textrm{O}}$, and $\sigma _{\textrm{O}}$ were treated as adjustable variables, whereas the gases parameters $\epsilon _{\textrm{C}}$, $\sigma _{\textrm{C}}$, $\epsilon _{\textrm{H}}$, $\sigma _{\textrm{H}}$, $\epsilon _{N}$, $\sigma _{N}$, $\epsilon _{O}$, and $\sigma _{O}$ were kept fixed at their initial values. This protocol preserves the substrate molecules description and assigns the fitting flexibility to the [MoO$_4$]$^{2-}$ anion, which is expected to be the least transferable fragment in the present nonbonded model. Parameter optimization was carried out with the L-BFGS-B algorithm under box constraints of 0.01–3.00 kcal/mol for ϵ and 1.5–6.0 Å for σ.

The optimization problem is formulated as the minimization of an objective function $f(\vec {p})$ with respect to the parameter vector $\vec {p}$:

7 $$\begin{aligned} \min _{\vec {p} \in B} f(\vec {p}) \end{aligned}$$

where $\vec {p} = [\sigma _{Mo}, \epsilon _{Mo}, \sigma _{O}, \epsilon _{O}]$ represents the set of four Lennard–Jones parameters to be optimized. The search space is constrained by the set of admissible values *B*, defined by lower and upper physical bounds:

8 $$\begin{aligned} B = {\vec {p} \mid p_{i}^{min} \le p_i \le p_{i}^{max}} \end{aligned}$$

To solve this constrained problem, we employed the L-BFGS-B algorithm. The algorithm updates the gradient estimate using a limited memory of pairs $(\vec {s}_k, \vec {y}_k)$, defined as:

9 $$\begin{aligned} \vec {s}_{k} = \vec {p}_{k}\,{+\,1} - \vec {p}_k, \quad \vec {y}_k = \nabla f(\vec {p}_{k}{\,+\,1}) - \nabla f(\vec {p}_k) \end{aligned}$$

The approximate Hessian matrix is constructed implicitly and applied at each iteration to search for the minimum of the objective function while respecting the imposed boundaries [53].

In the present work, the objective function combines the mean absolute deviation from the DFT reference energies across all configuration frames with a quadratic regularization term penalizing excessive deviations from the initial parameter set $p_0$ [54], as shown in Eq. 10:

10 $$\begin{aligned} F=\frac{1}{N}\sum _{k=1}^{N} \left| U_{\textrm{int},k} -\Delta U_{\textrm{DFT},k} \right| +\lambda \sum _{p}(p-p_0)^2 \end{aligned}$$

where λ, in Eq. 10, is the regularization coefficient that balances the fitting accuracy and parameter stability.

A particularly important aspect of the present strategy is that it provides a simple yet robust route to generating optimized nonbonded parameters for chemically distinct active sites without resorting to excessively complex fitting protocols. In the present formulation, only a small subset of physically meaningful LJ parameters is refined, while the remaining terms are kept fixed. The search space is constrained by explicit bounds, and the optimization target combines direct agreement with the reference interaction energies and a regularization term to prevent unphysical parameter drift. As a consequence, the procedure is computationally inexpensive, numerically stable, and straightforward to reproduce, while remaining flexible enough to capture the essential features of the quantum-mechanical interaction profile. This balance between simplicity and reliability is especially relevant for MOF-like materials, in which localized metal-containing sites often require system-specific parametrization but full force-field redevelopment is impractical. The protocol therefore offers a pragmatic framework for the rapid generation of optimized parameters for targeted adsorption sites, enabling the construction of improved classical models from a limited set of reference calculations. In this sense, the method is not intended as a highly elaborate global force-field optimization scheme, but rather as an efficient parametrization workflow for active-site refinement, precisely the level of description most needed for screening, comparative modeling, and mechanistic studies in porous materials. The intermolecular Lennard–Jones parametrization was carried out using a homemade code available at https://github.com/jcsdasilva/LJ-ParGen.git; the corresponding input file format is described in Section S4 of the Supplementary Information. To validate the optimized parameters beyond reproducing the interaction DFT energy curve, they were used to simulate the adsorption of CH$_4$, CO$_2$, and N$_2$ in MoOFOUR-1-Ni.

### GCMC adsorption simulations and IAST selectivity

Finally, the parameters were validated by comparison with adsorption isotherms simulated using the RASPA software [31]. The $2 \times 2 \times 2$ supercell employed in all simulations was derived from a unit cell of MoOFOUR-1-Ni optimized using periodic DFT calculations. The structural optimization was performed with the PBE functional [45] supplemented with Grimme’s D3 dispersion correction [55], under Periodic Boundary Conditions (PBC), as implemented in Quantum ESPRESSO (version 7.2) [56]. Valence electrons were described by a plane-wave basis set with a kinetic energy cutoff of 40 Ry, and core electrons were treated with Vanderbilt ultrasoft pseudopotentials [57]. Brillouin zone sampling was performed at the Γ point (1×1×1 Monkhorst–Pack grid) [58]. Both atomic positions and cell parameters were fully relaxed using the conjugate gradient method until forces converged below $1 \times 10^{-3}$ Ry Bohr$^{-1}$. The optimized lattice parameters are reported in Table S2. The resulting structure was then expanded into a $2 \times 2 \times 2$ supercell, whose lattice parameters are at least twice the van der Waals cutoff radius of 12 Å employed in the Grand Canonical Monte Carlo (GCMC) simulations, thereby satisfying the minimum image convention. The adsorption isotherms were computed via GCMC simulations in the μVT ensemble at 298 K, using $5 \times 10^4$ initialization cycles followed by $1 \times 10^6$ production cycles.

Three approaches were considered for describing the system: the UFF and Dreiding force fields [32, 33], the newly parameterized model for the molybdate anion, and experimental isotherms reported by Mohamed et al. [43], used as reference. Experimental adsorption data used for comparison were obtained by digitizing figures from the original published references using DataThief III [59]. Minor irregularities observed in the digitized isotherms, particularly in low-pressure regions where the adsorbed amount varies gradually, are attributed to the resolution of the source figures and do not reflect the physical behavior of the system. For the adsorbate molecules, the TraPPE force field parameters were employed [60].

To further assess the implications of the derived force field for the separation performance of MoOFOUR-1-Ni, the pure-component isotherms obtained from GCMC simulations were fitted to the dual-site Langmuir (DSL) model:

11 $$\begin{aligned} q(P) = \frac{m_1 b_1 P}{1 + b_1 P} + \frac{m_2 b_2 P}{1 + b_2 P} \end{aligned}$$

where $m_1$ and $m_2$ are the saturation capacities of each adsorption site (cm$^3$ g$^{-1}$) and $b_1$ and $b_2$ are the corresponding affinity constants (kPa$^{-1}$). For CH$_4$ and N$_2$, whose isotherms are quasi-linear over the pressure range studied, a single-site Langmuir expression was sufficient. The optimized DSL parameters are summarized in Table S6.

The fitted isotherms were subsequently employed as input to the Ideal Adsorbed Solution Theory (IAST) [61] to estimate binary adsorption selectivities for two industrially relevant gas mixtures: CO$_2$/CH$_4$ (50:50), representative of raw natural gas streams prior to liquefaction, and CO$_2$/N$_2$ (10:90), representative of post-combustion flue gas. The adsorption selectivity is defined as:

12 $$\begin{aligned} S\!\left( \frac{CO_2}{\text {X}}\right) = \frac{q_{CO_2}/q_{\text {X}}}{y_{CO_2}/y_{\text {X}}} \end{aligned}$$

where $q_i$ and $y_i$ denote the adsorbed amount and gas-phase mole fraction of component *i*, respectively.

**Fig. 1:**
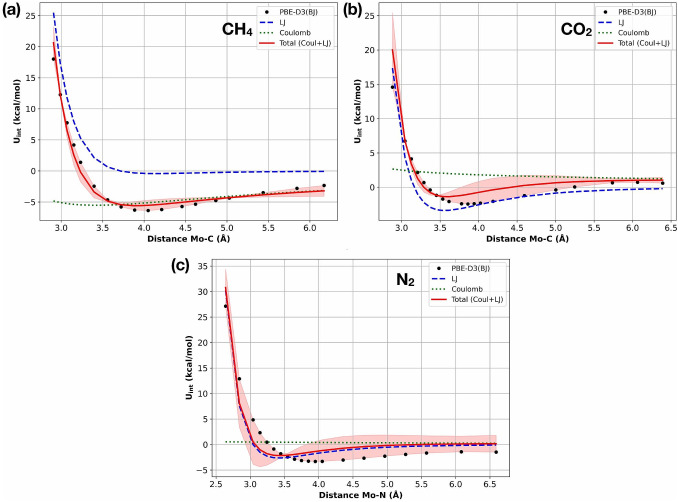
Combined interaction energy profiles for [MoO$_4]^{2-}$ with **a** CH$_4$, **b** CO$_2$, and **c** N$_2$. The plots show total energy (solid red line), Lennard–Jones contribution (dashed blue line), Coulombic contribution (dotted green line), and mean absolute errors (shaded red) compared to DFT reference data (black dots)

## Result and discussion

### Force field parametrization procedure

The interaction energy ($U_{int}$) profiles for the molybdate ion ([MoO$_4]^{2-}$) with CH$_4$, CO$_2$, and N$_2$ are presented in Fig. 1. These curves reveal fundamental differences in the nature of the non-bonded forces governing each system. The relative contributions of the Lennard–Jones and Coulombic potentials vary significantly depending on the chemical environment of the substrate.

As shown in Fig. 1a, the electrostatic contribution for the $[\text {MoO}_4]^{2-} \cdots \text {CH}_4$ system is attractive, providing a stabilizing component that complements the LJ potential to match the reference data. Conversely, for the CO$_2$ and N$_2$ complexes (Fig. 1b and c), the Coulombic term becomes repulsive due to the interaction between the partial negative charges localized on the nitrogen and oxygen atoms of the gases with the oxygen atoms of the molybdate ion. To compensate for this repulsion and reproduce the total DFT interaction energy, the optimization algorithm intensifies the LJ potential, resulting in a deeper potential well.

It is noteworthy that the mean absolute errors (MAE) for the $[\text {MoO}_4]^{2-} \cdots \text {CO}_2$ and $[\text {MoO}_4]^{2-} \cdots \text {N}_2$ systems are significantly higher than those observed for the methane complex. This discrepancy highlights the increased complexity of the interaction nature when the substrate possesses localized partial charges and a more heterogeneous electronic distribution. While CH$_4$ presents a relatively isotropic and non-polar environment, the quadrupolar character and the presence of electronegative centers in CO$_2$ and N$_2$ induce strong many-body effects and polarization that are not fully captured by the classical additive 12-6 Lennard–Jones model.

The optimized LJ parameters (ϵ and σ) for the molybdate ion interacting with CH$_4$, CO$_2$, and N$_2$ are summarized in Table 1; the complete set of initial and optimized parameters is provided in Table S5. The results exhibit significant variations across the three studied systems, directly reflecting the distinct physical nature of each interaction pair [36, 62]. This divergence highlights the high specificity required to accurately model the non-bonded interactions of the [MoO$_4]^{2-}$ ion within different chemical environments.

**Table 1:** Optimized Lennard–Jones parameters for the molybdate ion compared to original UFF values

Molecule	Atom Type	$\epsilon _{opt}$ (kcal/mol)	$\sigma _{opt}$ (Å)	$\epsilon _{UFF}$ (kcal/mol)	$\sigma _{UFF}$^a^ (Å)
CH$_4$	Mo	0.0100	2.778	0.056	2.534
	O$_{ion}$	0.0524	3.060	0.060	3.118
CO$_2$	Mo	0.3579	2.998	0.056	2.534
	O$_{ion}$	0.4702	2.839	0.060	3.118
N$_2$	Mo	0.0100	2.870	0.056	2.534
	O$_{ion}$	0.4985	3.377	0.060	3.118

The $\sigma _{UFF}$ values were obtained following the conversion $\sigma = 2^{-1/6}x_i$ to suit the Lennard–Jones type potential

These results underscore the challenges regarding the transferability of force field parameters. The optimized σ and ϵ parameters for the molybdate ion are found to be highly sensitive to the specific substrate, reflecting the specific interaction environment rather than universal atomic constants. For high-charge density ions like [MoO$_4]^{2-}$, parameter development must be carefully contextualized within the intended chemical environment to ensure physical reliability in molecular simulations.

To assess the validity of the derived parameters, their performance was evaluated through Grand Canonical Monte Carlo simulations of gas adsorption isotherms in the MOF MoOFOUR-1-Ni, whose primary binding site features a structural motif analogous to the molybdate ion.

### Adsorption isotherm validation

**Fig. 2:**
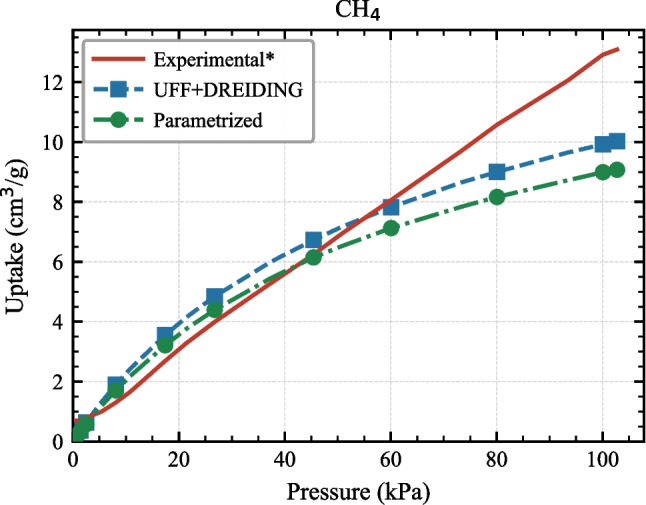
Comparison of simulated and experimental CH$_4$ adsorption isotherms on MoOFOUR-1-Ni at 298 K. Experimental data from Mohamed et al. [43]

The CH$_4$ isotherm is discussed first as it represents the simplest interaction case. As established in the interaction energy analysis, CH$_4$ presents an apolar, isotropic environment with a negligible quadrupole moment, resulting in weak interaction with the anionic molybdate sites of MoOFOUR-1-Ni. Consequently, both the UFF+DREIDING and reparameterized models yield similar uptake curves, with reasonable agreement with the experimental data across most of the pressure range, as shown in Fig. 2. The modest deviation observed at higher pressures, where the experimental isotherm exceeds both simulated curves, is attributed to limitations of the classical 12-6 Lennard–Jones repulsive term under dense packing conditions.

**Fig. 3:**
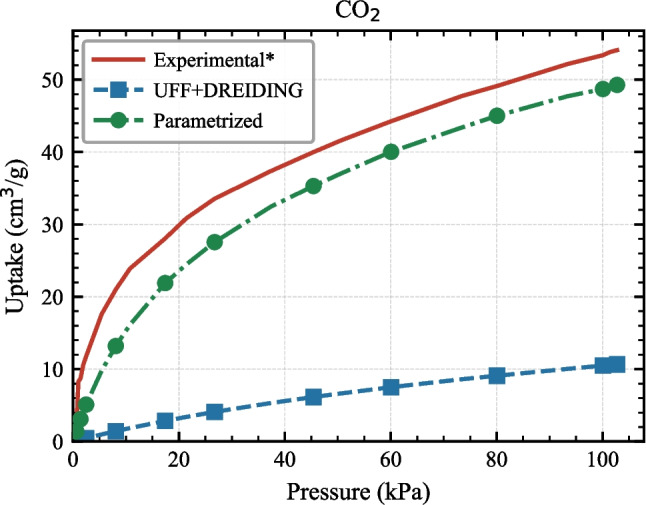
Comparison of simulated and experimental CO$_2$ adsorption isotherms on MoOFOUR-1-Ni at 298 K. Experimental data from Mohamed et al. [43]

As shown in Fig. 3, the UFF+DREIDING significantly underestimates CO$_2$ adsorption across the entire pressure range, consistent with its inability to capture the specific interaction environment of the molybdate anion. This behavior is consistent with the interaction energy analysis, where the repulsive Coulombic contribution, arising from the significant quadrupole moment of CO$_2$ ($-4.30\times 10^{-26} \text { esu} \, \text {cm}^{2}$), forces the LJ term to deepen the potential well, a balance that generic force fields fail to reproduce for the molybdate anion. The reparameterized model yields substantially improved agreement with the experimental isotherm, demonstrating that the optimized ϵ and σ parameters are transferable from the molecular interaction energy scale to the macroscopic adsorption regime.

**Fig. 4:**
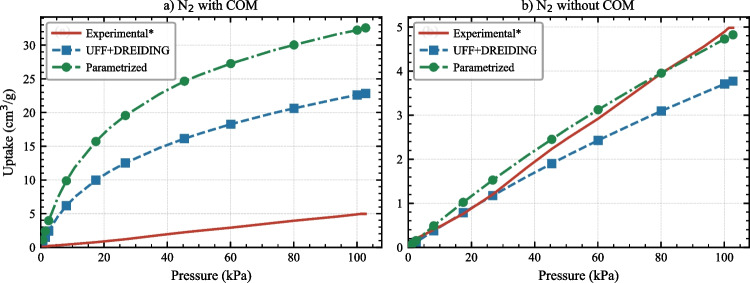
Comparison of simulated and experimental N$_2$ adsorption isotherms on MoOFOUR-1-Ni at 298 K. **a** N$_2$ modeled with an explicit center-of-mass (COM) charge site to account for its quadrupole moment. **b** N$_2$ treated as an apolar species, with the COM charge site omitted. Note the difference in uptake scale between panels. Experimental data digitized from Mohamed [43]

Finally, the most complex case is the N$_2$ system. As noted above, N$_2$ exhibits a small but non-negligible quadrupole moment ($-1.52 \times 10^{-26}$ esu cm$^2$), which is sometimes accounted for in classical simulations through an explicit charge site placed at the center of mass (COM). However, as shown in Fig. 4a, this representation leads to a significant overestimation of the adsorption capacity, a consequence of an artificially intensified anion–N$_2$ electrostatic interaction. Given that the choice of molecular representation is an integral part of classical force field development, an alternative approach was adopted in which the COM charge site was omitted, effectively treating N$_2$ as an apolar species. This approximation is physically motivated by the comparatively small quadrupole moment of N$_2$ ($-1.52 \times 10^{-26}$ esu cm$^2$) relative to CO$_2$ ($-4.30 \times 10^{-26}$ esu cm$^2$), together with the known limitations of fixed-charge non-polarizable force fields in describing highly localized electrostatic environments such as the [MoO$_4$]$^{2-}$ cavity. In the present case, the explicit COM representation appears to artificially overestimate the electrostatic attraction between N$_2$ and the anionic pillar, leading to adsorption capacities substantially larger than the experimental values. Omitting the COM charge site therefore provides an effective approximation that partially compensates for the absence of explicit polarization and many-body effects in the classical model. It should be noted, however, that this simplification may introduce non-negligible errors at higher pressures or under competitive adsorption conditions, where the quadrupolar character of N$_2$ may become more relevant. A more rigorous treatment would likely require either a polarizable force field or a dedicated recalibration of the quadrupolar representation specifically for this host environment.

**Table 2:** MAE and RMSE between simulated and experimental adsorption isotherms for each system and force field model

System	Model	MAE (cm$^3$/g)	RMSE (cm$^3$/g)
CH$_4$	UFF+DREIDING	1.04	1.41
	Parametrized	1.22	1.84
CO$_2$	UFF+DREIDING	27.06	30.10
	Parametrized	5.35	5.60
N$_2$ (with COM)	UFF+DREIDING	10.25	12.01
	Parametrized	16.28	18.95
N$_2$ (no COM)	UFF+DREIDING	0.38	0.59
	Parametrized	0.14	0.17

**Fig. 5:**
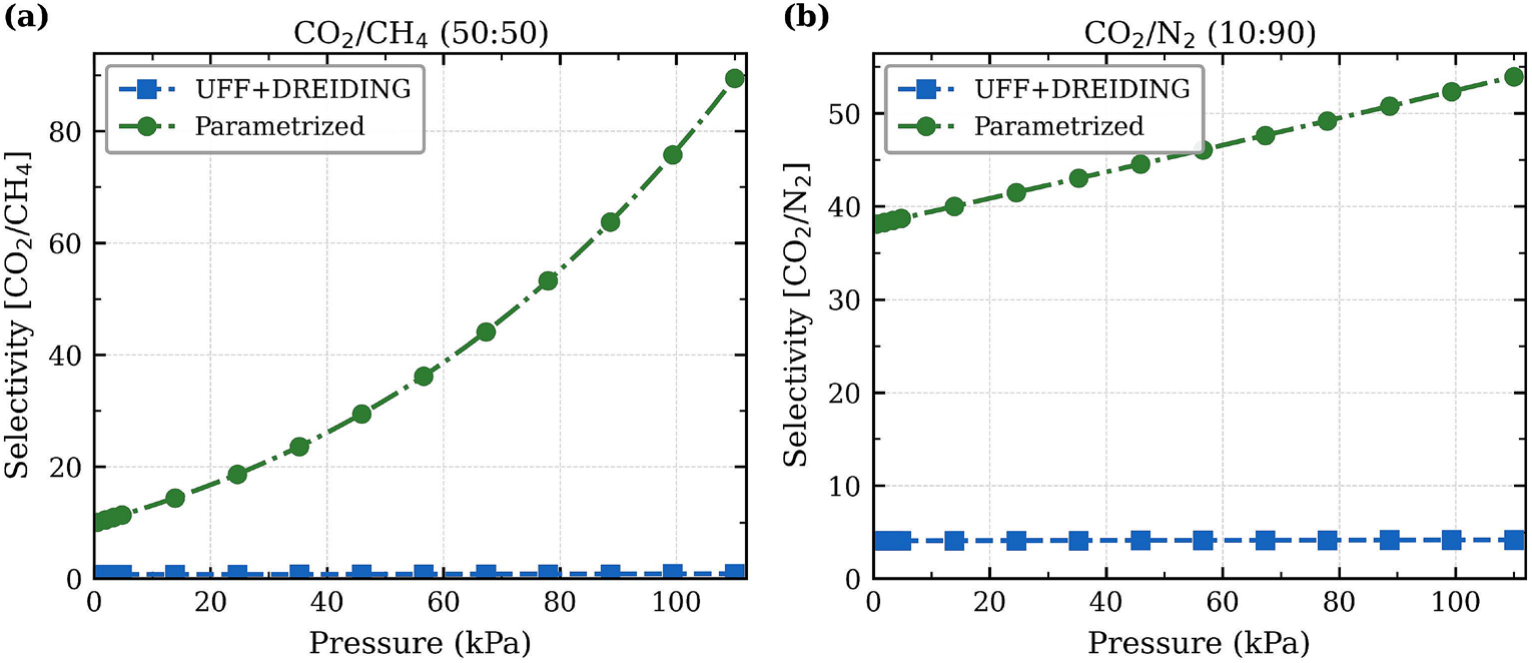
IAST adsorption selectivity of MoOFOUR-1-Ni at 298 K as a function of total pressure, predicted via DSL-fitted pure-component isotherms (Eq. 11). **a** CO$_2$/CH$_4$ equimolar mixture ($y_{CO_2} = y_{CH_4} = 0.50$). **b** CO$_2$/N$_2$ mixture at a typical post-combustion composition ($y_{CO_2} = 0.10$, $y_{N_2} = 0.90$). Blue squares and green circles correspond to the UFF+DREIDING and reparametrized force fields developed in this work

To provide a quantitative assessment of the agreement between simulated and experimental isotherms, the MAE and root mean square error (RMSE) were computed for each system and model, and the results are summarized in Table 2. For the CH$_4$ isotherm, the UFF+DREIDING and reparameterized models yield comparable errors (MAE = 1.04 and 1.22 cm$^3$/g, respectively), consistent with the weak and largely apolar character of the CH$_4$–molybdate interaction, for which generic parameters already provide a reasonable description. The detrimental effect of including the COM charge site for N$_2$ is also clearly reflected in the error metrics: both the UFF+DREIDING (MAE = 10.25 cm$^3$/g) and the reparameterized model (MAE = 16.28 cm$^3$/g) yield substantially larger errors when the quadrupole moment is explicitly represented, confirming that this representation artificially intensifies the anion–N$_2$ electrostatic interaction.

The most significant improvement is observed for CO$_2$, where the UFF+DREIDING reference produces a MAE of 27.06 cm$^3$/g and RMSE of 30.10 cm$^3$/g, reflecting its complete failure to capture the electrostatic environment of the molybdate anion. The reparameterized model reduces these errors to MAE = 5.35 cm$^3$/g and RMSE = 5.60 cm$^3$/g, representing an approximately five-fold improvement. For N$_2$ treated as an apolar species (no COM), the reparameterized model achieves MAE = 0.14 cm$^3$/g and RMSE = 0.17 cm$^3$/g, compared to MAE = 0.38 cm$^3$/g and RMSE = 0.59 cm$^3$/g for the UFF+DREIDING reference, demonstrating very good agreement with the experimental data across the full pressure range.

### IAST adsorption selectivity

The IAST selectivity curves for the CO$_2$/CH$_4$ and CO$_2$/N$_2$ mixtures are shown in Fig. 5. For the CO$_2$/CH$_4$ mixture (Fig. 5a), the reparametrized model predicts a strongly pressure-dependent selectivity, rising monotonically from approximately 10 at low pressure to 89 at 100 kPa. This increasing trend reflects the progressive saturation of CH$_4$ adsorption sites relative to the still-growing CO$_2$ uptake, a direct consequence of the deeper potential well imposed on the [MoO$_4$]$^{2-}$–CO$_2$ interaction by the optimized Lennard–Jones parameters. In sharp contrast, the UFF+DREIDING model yields $S(\text {CO}_2/\text {CH}_4) \approx 0.8$ throughout the entire pressure range—an unphysical inversion of selectivity implying preferential adsorption of CH$_4$ over CO$_2$. This result is a direct consequence of the near-coincidence of the UFF CO$_2$ and CH$_4$ isotherms at all pressures studied, which in turn stems from the failure of the generic force field to capture the strong electrostatic interaction between CO$_2$ and the molybdate pillar. Critically, this failure is not merely quantitative: a force field that predicts inverted selectivity would lead to qualitatively wrong conclusions about the viability of MoOFOUR-1-Ni as a CO$_2$/CH$_4$ separation material.

For the CO$_2$/N$_2$ mixture (Fig. 5b), the reparametrized model predicts selectivities in the range 38–54 over 10–100 kPa, with a modest positive pressure dependence consistent with the Henry’s-law-regime behavior of N$_2$ over this pressure window. The UFF+DREIDING reference yields $S(\text {CO}_2/\text {N}_2) \approx 4$ throughout the entire range—approximately one order of magnitude lower than the reparametrized estimate—once again reflecting the inability of the generic force field to reproduce the preferential affinity of the molybdate cavity for CO$_2$. While a direct experimental IAST benchmark is not available for these specific mixture compositions, the predicted selectivities from the reparametrized model are qualitatively consistent with the high CO$_2$ uptake relative to N$_2$ reported experimentally by [43].

Taken together, the IAST results demonstrate that errors in pure-component force fields propagate non-linearly into mixture separation predictions: the approximately five-fold improvement in CO$_2$ isotherm accuracy achieved by the reparametrized model translates into a qualitative correction of the predicted selectivity, recovering the physically expected preferential CO$_2$ adsorption behavior of MoOFOUR-1-Ni.

## Conclusion

In this work, we report the derivation of optimized Lennard–Jones parameters for the $[\text {MoO}_4]^{2-}$ ion using a context-specific fitting procedure based on DFT-derived potential energy surfaces. The results demonstrate that the resulting ϵ and σ parameters are highly sensitive to the chemical nature of the interacting substrate, with systems such as $\text {CO}_2$ and $\text {N}_2$ requiring substantially different values compared to methane to account for their localized partial charges and quadrupolar character properly. This pronounced dependence highlights the intrinsic limitations of transferable force field parameters in accurately describing chemically complex ionic environments.

Despite its conceptual simplicity, the adopted approach is based on a classical additive 12–6 Lennard–Jones model optimized via the L-BFGS-B algorithm and proved sufficiently robust to capture the essential features of the interaction landscape while maintaining computational efficiency. Although more advanced approaches, such as polarizable force fields or machine-learning-based potentials, may offer improved accuracy, the present strategy provides a practical and physically motivated alternative for generating system-specific parameters without incurring the substantial computational cost and methodological complexity associated with higher-level models.

Finally, the system-specific force field derived here provides a physically grounded foundation for molecular dynamics simulations of the MoOFOUR-1-Ni framework, enabling the direct computation of self-diffusion coefficients and the investigation of adsorption kinetics for CO$_2$, N$_2$, and CH$_4$ within the molybdate-lined pore channels, properties that lie beyond the reach of the static GCMC approach employed here.

## Supplementary Information

Below is the link to the electronic supplementary material.

Supplementary file 1 (pdf 229 KB)

## Data Availability

No datasets were generated or analysed during the current study.
