# Supplementary material for: DFT-guided Lennard–Jones parametrization for accurate CO2, N2, and CH4 adsorption in MoOFOUR-1-Ni
Source: J Mol Model. 2026 Aug 1;32(8):291. doi: 10.1007/s00894-026-06866-6 (PMC13428698; doi:10.1007/s00894-026-06866-6)
Supplement: Supplementary file 1 — (pdf 229 KB) [file 894_2026_6866_MOESM1_ESM.pdf]

Supplementary Information
DFT-Guided Lennard-Jones Parametrization for Accurate CO_2, N_2, and CH_4 Adsorption in MoOFOUR-1-Ni

Herick Ribeiro Torres[1], Roberta Pereira Dias[2],
Heitor Avelino de Abreu[1], Júlio Cosme Santos da Silva[2*],
Guilherme Ferreira de Lima[1*]

[1]Departamento de Química, Universidade Federal de Minas Gerais, Av. Antônio Carlos, 6627, Pampulha, Belo Horizonte, 31270-901, MG, Brazil.
[2]Instituto de Química e Biotecnologia, IQB, Universidade Federal de Alagoas, Campus A. C. Simões, Maceió, 57072-900, Al, Brazil.

*Corresponding author(s). E-mail(s): julio.silva@iqb.ufal.br; gflima@ufmg.br;

1 S1 - CHELPG Partial Charges for the $[MoO_4]^{2-}$ Anion

Table 1 CHELPG partial charges (in units of elementary charge e) for the $[MoO_4]^{2-}$ anion computed in the presence of each gas molecule. The total charge of the anion is conserved at $-2\,e$ in all three systems.

Atom	CH_4 system	CO_2 system	N_2 system
Mo	+2.2405	+2.2325	+2.2468
O_1	−1.0680	−1.0577	−1.0648
O_2	−1.0490	−1.0156	−1.0351
O_3	−1.0477	−1.0055	−1.0317
O_4	−1.0478	−1.0090	−1.0309

2 S2 - Lattice Parameters

Table 2 Experimental and theoretically optimized lattice parameters of the reduced unit cell of MoOFOUR-1-Ni. Experimental values from Mohamed *et al.* [1].

Parameter	Exp.	Theo.	Δ (%)
a (Å)	13.5673	13.691	0.91
b (Å)	13.5673	13.691	0.91
c (Å)	13.5673	13.696	0.95
α (°)	101.9870	102.3	0.31
β (°)	101.9870	102.4	0.40
γ (°)	101.9870	102.4	0.40
V (Å^3)	2306.071	2356	2.17

3 S3- Representative ORCA Input for DFT Interaction Energy Calculations

The input file below illustrates the protocol used to generate the potential energy curves for the $[MoO_4]^{2-}$–CO_2 system. The molybdate anion was kept fixed at the origin of the coordinate system, with the molybdenum atom placed at the center. The intermolecular distance R is defined along the x-axis as the separation between the Mo atom and the carbon atom of CO_2. Single-point energy calculations were performed for 50 evenly spaced values of R in the range 2.39–6.39 Å, while all internal geometries were kept frozen. Analogous inputs were employed for the CH_4 and N_2 systems, with R defined with respect to the carbon atom and one of the nitrogen atoms, respectively.

```
%Pal nprocs 4 end
%MaxCore 400
! PBE D3BJ def2-TZVPD VeryTightSCF
%paras
  R= 2.3893145165, 6.3893145165, 50
end
*xyz -2 1
Mo    0.0001380000    0.0000490000    0.0001650000
O    -1.7685840000    0.0172430000    0.0890030000
O     0.5038250000   -0.5220690000   -1.6153320000
O     0.6233640000    1.6272550000    0.3155120000
O     0.6409390000   -1.1212520000    1.2112960000
C    {R}             -0.0357647766   -0.1250482818
O    {R-0.003149172}  1.1319944966   -0.2167711937
O    {R+0.003017599} -1.2035331833   -0.0333764078
*
```

The `%paras` block defines R as a parametric variable ranging from 2.39 to 6.39 Å across 50 steps, which ORCA evaluates sequentially in a single run. The total charge of -2 and multiplicity of 1 correspond to the isolated $[MoO_4]^{2-}$ anion with the neutral CO_2 molecule.

4 S4 - LJ_Par Input Model

```
NF: Frame number
NA: number of atoms in fragment A
NB: number of atoms in fragment B
ATA: Reference atom to scan in fragment A
ATB: Reference atom to scan in fragment B
Uint: DFT interation energy (kcal/mol)

Input Model Format

NF
NA NB ATA ATB Uint
Symbol X  Y  Z Atomic_charge Epsilon(initial) Sigma(initial)

Example:

1
5 5 1 6 18.01
Mo  0.000138  0.000049  0.000165  2.11 0.060 2.72
O  -1.768584  0.017243  0.089003 -1.03 0.105 3.30
O   0.503825 -0.522069 -1.615332 -1.08 0.105 3.30
O   0.623364  1.627255  0.315512 -1.08 0.105 3.30
O   0.640939 -1.121252  1.211296 -1.07 0.105 3.30
C   2.903350  0.154472 -0.004246 -1.00 0.095 3.47
H   2.903350 -0.566833  0.710279  0.14 0.015 2.84
H   1.903350  0.076856 -0.114982  0.74 0.015 2.84
H   2.903350 -0.029633 -0.947017  0.09 0.015 2.84
H   2.903350  1.137496  0.334733  0.18 0.015 2.84

2
5 5 1 6 12.28
Mo  0.000138  0.000049  0.000165  2.11 0.060 2.72
O  -1.768584  0.017243  0.089003 -1.03 0.105 3.30
O   0.503825 -0.522069 -1.615332 -1.08 0.105 3.30
O   0.623364  1.627255  0.315512 -1.08 0.105 3.30
O   0.640939 -1.121252  1.211296 -1.07 0.105 3.30
C   2.984983  0.154472 -0.004246 -1.00 0.095 3.47
H   2.984983 -0.566833  0.710279  0.14 0.015 2.84
H   1.984983  0.076856 -0.114982  0.74 0.015 2.84
H   2.984983 -0.029633 -0.947017  0.09 0.015 2.84
H   2.984983  1.137496  0.334733  0.18 0.015 2.84
```

5 S5 - Initial and Optimized Lennard-Jones parameters for the molybdate ion

Table 3 Initial and Optimized Lennard-Jones parameters for the molybdate ion.

Molecule	Atom Type	$\epsilon_{initial}$ (kcal/mol)	$\sigma_{initial}$ (Å)	ϵ_{opt} (kcal/mol)	σ_{opt} (Å)
CH_4	Mo	0.060	2.720	0.0100	2.279
	O_{ion}	0.105	3.30	0.026	3.272
CO_2	Mo	0.060	2.720	0.357	2.998
	O_{ion}	0.105	3.30	0.4702	2.839
N_2	Mo	0.060	2.720	0.0100	2.870
	O_{ion}	0.105	3.300	0.4985	3.377

6 S6 - DSL Parameters

Table 4 Dual-site Langmuir parameters fitted to the pure-component adsorption isotherms of CO_2, CH_4, and N_2 in MoOFOUR-1-Ni at 298 K. For CH_4 and N_2 a single-site Langmuir model was employed ($m_2 = 0$).

Gas	Model	m_1 (cm^3 g^{-1})	b_1 (kPa^{-1})	m_2 (cm^3 g^{-1})	b_2 (kPa^{-1})
CO_2	UFF+DREIDING	13.4	1.19×10^{-2}	500.0	7.0×10^{-5}
	Parametrized	64.2	5.12×10^{-3}	31.3	6.42×10^{-2}
CH_4	UFF+DREIDING	16.0	1.62×10^{-2}	—	
	Parametrized	14.4	1.63×10^{-2}	—	
N_2	UFF+DREIDING	17.2	2.74×10^{-3}	—	
	Parametrized	20.2	3.05×10^{-3}	—	

References

[1] Mohamed MH, Elsaidi SK, Wojtas L, et al (2012) Highly selective co2 uptake in uninodal 6-connected “mmo” nets based upon mo42−(m= cr, mo) pillars. Journal of the American Chemical Society 134(48):19556–19559
